# Missing and Delayed Auditory Responses in Young and Older Children with Autism Spectrum Disorders

**DOI:** 10.3389/fnhum.2014.00417

**Published:** 2014-06-06

**Authors:** J. Christopher Edgar, Matthew R. Lanza, Aleksandra B. Daina, Justin F. Monroe, Sarah Y. Khan, Lisa Blaskey, Katelyn M. Cannon, Julian Jenkins, Saba Qasmieh, Susan E. Levy, Timothy P. L. Roberts

**Affiliations:** ^1^Department of Radiology, Lurie Family Foundation MEG Imaging Center, The Children’s Hospital of Philadelphia, Philadelphia, PA, USA; ^2^Department of Pediatrics, The Children’s Hospital of Philadelphia, Philadelphia, PA, USA

**Keywords:** autism spectrum disorders, M50, M100, superior temporal gyrus, magnetoencephalography

## Abstract

**Background:** The development of left and right superior temporal gyrus (STG) 50 ms (M50) and 100 ms (M100) auditory responses in typically developing (TD) children and in children with autism spectrum disorder (ASD) was examined. Reflecting differential development of primary/secondary auditory areas and supporting previous studies, it was hypothesized that whereas left and right M50 STG responses would be observed equally often in younger and older children, left and right M100 STG responses would more often be absent in younger than older children. In ASD, delayed neurodevelopment would be indicated via the observation of a greater proportion of ASD than TD subjects showing missing M100 but not M50 responses in both age groups. Missing M100 responses would be observed primarily in children with ASD with language impairment (ASD + LI) (and perhaps concomitantly lower general cognitive abilities).

**Methods:** Thirty-five TD controls, 63 ASD without language impairment (ASD − LI), and 38 ASD + LI were recruited. Binaural tones were presented. The presence or absence of a STG M50 and M100 was scored. Subjects were grouped into younger (6–10 years old) and older groups (11–15 years old).

**Results:** Although M50 responses were observed equally often in older and younger subjects and equally often in TD and ASD, left and right M50 responses were delayed in ASD − LI and ASD + LI. Group comparisons showed that in younger subjects M100 responses were observed more often in TD than ASD + LI (90 versus 66%, *p* = 0.04), with no differences between TD and ASD − LI (90 versus 76%, *p* = 0.14) or between ASD − LI and ASD + LI (76 versus 66%, *p* = 0.53). In older subjects, whereas no differences were observed between TD and ASD + LI, responses were observed more often in ASD − LI than ASD + LI. Findings were similar when splitting the ASD group into lower- and higher-cognitive functioning groups.

**Conclusion:** Although present in all groups, M50 responses were delayed in ASD. Examining the TD data, findings indicated that by 11 years, a right M100 should be observed in 100% of subjects and a left M100 in 80% of subjects. Thus, by 11 years, lack of a left and especially right M100 offers neurobiological insight into sensory processing that may underlie language or cognitive impairment.

## Introduction

Autism spectrum disorders (ASD) are a set of developmental disorders characterized by social impairments, stereotypical behaviors, and deficits in communication. As a childhood disorder, an understanding of brain abnormalities in ASD requires an examination of brain processes in infants, toddlers, and young and older school-aged children with ASD. A growing number of electroencephalography (EEG) and magnetoencephalography (MEG) studies report auditory abnormalities in children with ASD. Findings include delayed superior temporal gyrus (STG) auditory 100 ms responses in children with ASD (Roberts et al., 2010), reduced 40 Hz auditory steady-state total power in children with ASD (Wilson et al., 2007), pre- and post-stimulus pure tone STG abnormalities in children with ASD (Edgar et al., 2013), and atypical hemispheric lateralization of auditory responses in children with ASD (Stroganova et al., 2013).

As reviewed below, EEG and MEG studies examining auditory processes in children with ASD differ from adult EEG and MEG studies, with some auditory components observed at a longer latency in children than adults, and some components more likely to be observed in children and not in adults, and vice versa. Building upon previous studies investigating the development of auditory responses in typically developing (TD) children (e.g., Ponton et al., 2002), the present study examined the development of STG auditory responses in children with ASD to determine if there was evidence for a developmental delay.

The text below reviews the literature on auditory responses in adults and children. Given that a primary goal of the present study is the use of MEG to examine left and right STG auditory responses in children with ASD, the tangential auditory responses best measured using MEG are a primary focus. [For EEG studies examining the development of auditory components due to radially oriented neurons on the lateral aspect of the STG, generally not detected using MEG, readers are directed to Ponton et al. (1999) and Ponton et al. (2002) and also to EEG studies examining these radially oriented auditory components in ASD (Bruneau et al., 1997; Orekhova et al., 2009; Stroganova et al., 2013)].

### Auditory responses in adults

In adults, N100 (EEG) and M100 (MEG) are the most prominent deflections of the auditory event-related potential (EEG) or field (MEG), evolving with a peak latency of about 100 ms after stimulus onset (for a review see Hari, 1990). Näätänen and Picton (1987) argued that the electric N100 reflects contributions from five to six distinct cortical areas: dipoles in or near the primary auditory cortex, a frontal source, and early portions of the attention-related processing negativity and the mismatch negativity. Using BESA and VARETA to model the N100 sources, Picton et al. (1999) noted that although multiple brain regions contribute to N100, the major activity underlying the scalp-recorded N100 wave is located in the supratemporal plane. Because MEG does not detect activity from radial current configurations, M100 is well described as being generated by a pair of equivalent current dipoles (one in each hemisphere) located in the region of the planum temporale (e.g., Hari, 1990; Lutkenhoner and Steinstrater, 1998).

In adults, a smaller auditory response around 50 ms (EEG P1 or P50 and MEG M50) is also often seen. The relevant MEG literature points to STG as the M50 generator (e.g., Pelizzone et al., 1987; Reite et al., 1988; Mäkelä et al., 1994; Yoshiura et al., 1995; Huotilainen et al., 1998; Yvert et al., 2001). Investigators using either intraoperative electrocorticography (Ligeois-Chauvel et al., 1994) or chronic subdural electrodes (Lee et al., 1984) have reported that P50 is a near-field potential in the primary auditory cortex. The supratemporal origin is also supported by the scalp distribution of electrical potentials (Cohen, 1982) and by recordings from the pial surface over temporal and parietal lobes (Chatrian et al., 1960). Although it has been suggested that areas such as hippocampus (Goff, 1978; Waldo et al., 1994; Freedman et al., 1996), midbrain reticular (Erwin and Buchwald, 1986a,b), and midline brain regions (Kraus et al., 1992; Ninomiya et al., 1997) contribute to the scalp-recorded 50 ms response, the contribution from these non-STG sources is likely small. As an example, predicting EEG P50 Cz scalp-recorded activity from bilateral STG sources derived from whole-cortex MEG, Huang et al. (2003) showed that virtually all of the variance in P50 Cz in adult controls (96%) is accounted for by STG sources. In terms of the number of sources that could possibly contribute to EEG P50, the work by Grunwald et al. (2003) is also relevant, as they recorded directly from cortex. Examining 1270 subdural contacts, they did not observe 50 ms activity in the majority of cortical recording sites, concluding that “P50 is not generated in widespread cortical areas.”

### Auditory responses in children

Auditory responses in children differ from those observed in adults, with P50 more readily evoked in young children, and with P50 (P1) peak latency in children 5–6 years of age ~85–95 ms (Wunderlich and Cone-Wesson, 2006). P50 latency and amplitude decrease as a function of age (e.g., Paetau et al., 1995). Ponton et al. (2002) and Ceponiene et al. (2002) suggest that the attenuation in P50 amplitude as a function of age arises from the phase cancelation of the later parts of the P50 peak by the increasing magnitude of the N100 neural generators. Although less common in young children, when present, N100 appears around 100–150 ms (e.g., Satterfield et al., 1984; Bruneau et al., 1997; Ponton et al., 2000).

In older children, auditory responses become more complex and the components more defined, with an adult morphology typically observed around 10–12 years of age (Ponton et al., 2000), and thus with EEG N100 and MEG M100 auditory responses generally observed by late childhood and early adolescence (Ponton et al., 2000, 2002). There is conflicting evidence as to the effect of age on N100 amplitude. Some studies show that N100 amplitude increases with age (Bruneau et al., 1997; Ponton et al., 2000, 2002), whereas others have found no effect (e.g., Tonnquist-Uhlén, 1996; Ceponiene et al., 2002). Inter-stimulus interval (ISI) may account for study differences – in children, N100 generators require longer intervals to produce a large response given longer refractory periods in children than adults (e.g., Paetau et al., 1995; Rojas et al., 1998). The amplitude of a response occurring after the N100 and with the same general topography, N200, has an amplitude that is constant up to age 11 and then decreases (Ponton et al., 2002).

### Missing auditory responses in children with ASD

In a preliminary report examining the presence or absence of left and right STG M100 responses, using a 1000 ms inter-trial interval, Khan et al. (2010) observed that M100 responses were observed more frequently in TD subjects than children with ASD. In particular, M100 responses were deficient especially in children with concomitant language impairment, and especially in the left-hemisphere. The present study further examined the occurrence of STG M50, M100, and M200, examining left and right STG responses, examining a larger population of TD and ASD children, and examining STG responses at a longer inter-trial interval (average 2,350 ms) to increase the possibility of observing auditory responses in young children. In addition, analyses were expanded to determine whether the “missing” M100 responses were unique to language impairment or were a pattern also observed in children with general intellectual impairments as indicated by lower IQ.

The following hypotheses were made:

1. Reflecting differential development of primary/secondary auditory cortex areas and supporting previous studies (e.g., Rojas et al., 1998; Yoshimura et al., 2012), whereas left and right M50 STG responses would be observed equally often in younger and older children, left and right M100 STG responses would more often be absent in younger than older children.
2. In ASD, delayed neurodevelopment would be indicated via the observation of a greater proportion of ASD than TD subjects showing missing M100 but not M50 responses in both age groups. Missing M100 responses would be observed primarily in children with ASD with language impairment (ASD + LI) (and perhaps concomitantly lower general cognitive abilities).

## Materials and Methods

### Subjects

Diagnoses of ASD were determined prior to recruitment based on the child’s performance during clinical interviews, their documentation of DSM-IV criteria for ASD, and results from tests such as the Childhood Autism Rating Scale and the autism diagnostic observation schedule (ADOS). Advertisements through local newspapers and pediatric practices within the Children’s Hospital of Philadelphia (CHOP) primary care network were utilized for recruitment of TD controls.

The subjects’ first session at CHOP included clinical and diagnostic neuropsychological testing by a licensed child psychologist with expertise in autism (Lisa Blaskey) to ensure that all subjects met the minimum criteria for inclusion and to further confirm diagnoses of ASD, particularly by utilizing the ADOS, Social Responsiveness Scale (SRS), Krug Asperger’s Disorder Index (KADI), and Social Communication Questionnaire (SCQ). For confirmation of the ASD diagnosis, all children had to exceed established cut-offs on both the ADOS and SCQ. Subjects one point below cut-off for ADOS scores were permitted entry into the study as long as they exceeded cut-offs on at least two parent questionnaires. In the rare event that diagnosis could not be confirmed via use of the ADOS and parent questionnaires alone, the autism diagnostic interview-revised (ADI-R) was administered to provide final clarification of diagnosis.

For testing language impairment, all subjects were evaluated with the clinical evaluation of language fundamentals – fourth edition (CELF-4). The ASD sample was divided into ASD without language impairment (ASD − LI) and ASD + LI groups. The ASD + LI group included subjects scoring at or below the 16th percentile (SS < 85) on the CELF-4 Core Language Index. All subjects scored at or above the 5th percentile (SS > 75) on the perceptual reasoning index (PRI) of the Wechsler Intelligence Scale for Children-IV (WISC-IV). The WISC-IV verbal comprehension index (VCI) was also obtained.

The total number of subjects included 35 TD and 101 ASD (63 ASD − LI, 38 ASD + LI). Although analyzing data from a different task, the present sample includes several, although not all, of the subjects reported in Roberts et al. (2010) (17 TD and 25 ASD). The study was approved by the CHOP IRB and all participants’ families gave written consent.

### Auditory stimuli

Stimuli consisted of 1000 and 2000 Hz tones presented using Eprime v1.1. Tones were presented via a sound pressure transducer and sound conduction tubing to the subject’s peripheral auditory canal via ear-tip inserts (ER3A, Etymotic Research, IL, USA). Prior to data acquisition, 1000 Hz tones of 300 ms duration and 10 ms rise time were presented binaurally and incrementally until reaching auditory threshold for each ear. Tones were presented at 45 dB sensation level above threshold. Each trial consisted of a 50 ms tone (*S*_1_; randomly presented 1000 and 2000 Hz tones), an 800 ms ISI, a second 50 ms tone (*S*_2_; randomly presented 1000 and 2000 Hz tones), and a 2350 ms (±100) inter-trial interval. A total of 120 tone pairs were presented. The present study reports on *S*_1_ – the auditory response occurring after the long inter-trial interval and thus with the greatest recovery period. To obtain a response with sufficient trials, the *S*_1_ average included 1000 and 2000 Hz tones.

### MEG recordings

Magnetoencephalography data were obtained using a whole-cortex 275-channel system (VSM MedTech Inc., Coquitlam, BC, USA) in a magnetically shielded room. Prior to data acquisition, three head-position indicator coils were attached to the subject’s scalp at the nasion, left-, and right-preauricular points, providing continuous specification on head position and orientation in relation to the MEG sensors. A movie (without sound) was displayed to prevent fatigue.

Electrodes were attached to the left and right clavicles for electrocardiogram recordings (ECG) and to the bipolar oblique (upper and lower left sites) for electro-oculogram recordings (EOG). A band-pass filter (0.03–150 Hz) was placed over the EOG, ECG, and MEG signals, which were then digitized at 1200 Hz with third order gradiometer environmental noise reduction over the MEG data.

### MEG data analysis

Artifact correction was applied to remove eye-blink and cardiac activity (see Roberts et al., 2010) using BESA 5.2™. Epochs with artifacts other than blinks and heartbeats were rejected on the basis of amplitude and gradient criteria (amplitude >1200 fT/cm, gradients >800 fT/cm/sample). Artifact-free epochs (1000 + 2000 Hz tones) were then averaged according to stimulus type and filtered using a 1 Hz (6 dB/octave, forward) to 40 Hz (48 dB/octave, zero-phase) band-pass. Although group differences in the number of artifact-free trails were observed, *F*(2, 133) = 8.82, *p* < 0.001, the difference between groups in the mean number of trials was small: TD mean of 110 trials (range 83–119) = ASD − LI mean of 107 trials (range 86–119) > ASD + LI mean = 101 trials (range 81–116).

The presence or absence of M50, M100, and M200 responses in the left and right STG was determined by applying a standard source model transforming the raw MEG surface activity into brain space (MEG data co-registered to the Montreal Neurologic Institute averaged brain) using a model with multiple sources (Scherg and Berg, 1996). Specifically, the source model included left and right STG regional sources positioned at Heschl’s gyrus and nine fixed regional sources modeling brain background activity and acting as probe sources for additional activity. Each subject’s eye-blink and heartbeat source vectors were included in the individual source models (Berg and Scherg, 1994).

To optimize the orientation of the standard STG sources, the left- and right-hemisphere dipoles were oriented at the maximum of the M100. Presence of a M100 was determined based on amplitude, latency, and hemisphere ingoing and outgoing flux topography (e.g., left-hemisphere ingoing anterior, outgoing posterior, and vice versa for the right-hemisphere). In particular, a M100 was scored if the magnetic flux topography were characteristic of the M100 response, was preceded by M50 (i.e., flux topography opposite M100), and followed by M200 (i.e., flux topography same as M100), and with source strength greater than baseline. In the present study, M50 was operationally defined as the first reversal in magnetic-field topography preceding M100 (or M200 if M100 not present). As reported below, in many subjects, a left- or right-hemisphere M100 response was not observed. For these subjects, left and right STG dipoles were oriented at the maximum of M50. If neither a M50 nor M100 was observed, the dipole was oriented at M200. Identification of auditory responses and orientation of the STG dipoles were done blind to group.

Of the subjects examined, 18 subjects (4 TD, 7 ASD − LI, 7 ASD + LI) did not have observable M50, M100, or M200 responses in the left- or right-hemisphere. Lack of an observable auditory response in these subjects was due to large metal artifact or poor compliance. Data from these subjects was excluded. Examining only the subjects with an identifiable auditory response, goodness-of-fit values (average from the start of M50 to M200) did not differ between the TD and ASD groups [TD = 94% (SD = 2.28), ASD = 93% (SD = 3.00); *p* > 0.05]. Examining all subjects, maximum head motion during the recording was greater in younger than older subjects [young = 1.5 cm (SD = 1.2), older = 0.92 (SD = 0.92), *p* < 0.01].

In subjects with usable data (with either a 50, 100, and 200 ms response and thus with clear evidence that the subject heard the tone), epochs were defined from the continuous recording: 500 ms before the first tone to 500 ms after the first tone. When a M50 or M100 response was observed, left and right M50 (35–120 ms) and M100 (80–185 ms) latency data were calculated from the largest point in the scoring windows using in-house MATLAB software after subtraction of prestimulus baseline activity. Given that in many subjects the M200 was of long duration and without a clear peak, M200 responses were simply scored as present or absent, with M200 defined as a sustained response occurring after the M100 interval (i.e., 80–185 ms) and showing a magnetic-field topography similar to M100.

### Statistical analyses

Using IMB SPSS Statistics 20, *t*-tests examined group differences in age, CELF-4 scores, SRS, and IQ scores. For between-subject analyses, chi-square tests examined group differences in the presence or absence of a M50, M100, and M200 response (where an individual cell count was five or less, the Fisher Exact Test was used). For within-subject analyses, McNemar tests were used (McNemar, 1947). For analyses examining age differences, a median split separated subjects into younger (6–10 years old) and older (11–15 years old) groups. Finally, to examine if any ASD − LI versus ASD + LI missing M100 findings were specific to language impairment, using the PRI scores, the ASD group was also divided into a low and high IQ group (median split) and analyses re-run.

In the subset of subjects with a M50 or M100 response, repeated measures ANOVA examined group (TD, ASD − LI, ASD + LI), hemisphere, and group × hemisphere differences in latency. To examine how M50 and M100 latency differs as a function of group and age, hierarchical regression was run entering age first, diagnosis second, and their interaction last, with M50 or M100 latency as the dependent variable.

## Results

As shown in Table 1, groups did not differ in age. As expected, TD and ASD − LI subjects had significantly higher VCI and CELF-4 core language index scores than the ASD + LI subjects. As shown in Table 1, individuals with ASD − LI had higher PRI scores than individuals with ASD + LI.

**Table 1:** **Age, language, and cognitive information for each group (means and SD), (a) comparing younger TD to ASD (total sample), (b) comparing older TD to ASD (total sample), (c) comparing younger ASD − LI to ASD + LI, (d) comparing older ASD − LI to ASD + LI**.

Groups	Meanh	SD	Mean	SD	Groups	Mean	SD	Mean	SD
(a) Younger	TD (*N* = 20)		ASD (*N* = 79)		(b) Older	TD (*N* = 16)		ASD (*N* = 34)
Age	8.79	1.52	8.53	1.22	Age	13.46	1.58	13.07	1.33
PRI	108.95	15.5	104.37	16.98	PRI	108.75	12.94	102.88	14.94
VCI**	109.35	14.91	96.43	18.18	VCI	104	12.65	98.88	16.84
CELF**	109.36	12.41	86	21.74	CELF**	108.88	9.28	90.69	19.16

**Groups**	**Mean**	**SD**	**Mean**	**SD**	**Groups**	**Mean**	**SD**	**Mean**	**SD**
**(c) Younger**	**ASD − LI (*N* = 45)**		**ASD + LI (*N* = 34)**		**(d) Older**	**ASD − LI (*N* = 23)**		**ASD + LI (*N* = 11)**

Age	8.74	1.25	8.26	1.15	Age	13.23	1.32	12.73	1.34
PRI**	110.84	16.09	95.79	14.26	PRI**	109.26	11.29	89.55	12.93
VCI**	107.69	11.19	81.53	14.59	VCI**	107.7	11.87	80.45	8.58
CELF**	101.26	10.87	65.35	14.37	CELF**	101.38	10.89	67.36	10.1

**p < 0.01.

*As noted in the Section “Materials and Methods,” a median split separated subjects into younger (6–10 years old) and older (11–15 years old) groups*.

### M50

Collapsing across group and hemisphere, M50 responses were observed equally often in older (88.3%) and younger subjects (92.6%; *p* = 0.27). Collapsing across group and age, the presence or absence of M50 differed between the right (85.4%) and left-hemisphere (94.2%; McNemar *p* = 0.02). Given a significant difference between hemisphere but not age, simple effect analyses examined group differences for each hemisphere, collapsing across age group.

In the left-hemisphere, collapsing across age, no differences were observed between TD (91.7%) and ASD + LI (92.1%; Fisher *p* = 1.0), TD (91.7%) and ASD − LI (96.8%; Fisher *p* = 0.35), or ASD − LI (96.8%) and ASD + LI (92.1%; Fisher *p* = 0.36). In the right-hemisphere, collapsing across age, no differences were observed between TD (88.9%) and ASD + LI (81.6%; Fisher *p* = 0.52), TD (88.9%) and ASD − LI (85.7%; Fisher *p* = 0.76), or ASD − LI (85.7%) and ASD + LI (81.6%; *p* = 0.58). Splitting at the median head motion value, within each diagnostic age group, chi-square analyses showed that the presence or absence of M50 did not differ between individuals with more versus less head motion.

Table 2 (left column) shows the likelihood of observing a M50 for each group as a function of hemisphere. The Supplementary Material shows the likelihood of observing a M50 response in each group at each age.

**Table 2:** **Likelihood of observing a M50, M100, and M200 for each group as a function of age (young, older) for the left-hemisphere (left), right-hemisphere (right), and the average of the left- and right-hemisphere (average)**.

	*N*	M50			M100			M200		
		Left (%)	Right (%)	Average (%)	Left (%)	Right (%)	Average (%)	Left (%)	Right (%)	Average (%)
**TD**										
Young	20	84.2	94.7	89.5	90	95	92.5	100	100	100
Old	16	81.3	100.0	90.6	93.75	81.25	87.5	93.75	93.75	93.75
**ASD − LI**										
Young	42	73.8^a^	78.6	76.2^a^	95.24	78.57^a^	86.9^a^	100	95.24	97.6
Old	21	95.2^a^	95.2	95.2^a^	100	100^a^	100^a^	100	100	100
**ASD + LI**										
Young	28	60.7	75.0	66.1	96.43	78.57	87.5	100	96.48	98.2
Old	10	70.0	80.0	75.0	80	90	85	80	90	85

*For M50, Fisher exact tests showed an effect of age only in ASD − LI (average young = 76.2%, older = 95.2%; Fischer *p* = 0.01)*.

*^a^ For M100, although a main effect of group was observed, Fisher tests showed an effect of age only in ASD − LI (average young = 86.9%, older = 100%, *p* = 0.02)*.

*For M200, Fisher exact tests showed a significant effect of age in ASD + LI (average young = 98.2%, older = 85%, *p* = 0.05)*.

### M100

Collapsing across group and hemisphere, M100 responses were observed more often in older (89.4%) than younger subjects (77.5%; *p* = 0.02). Collapsing across group and age, the presence or absence of M100 did not differ between the right (85%) and left-hemisphere (77.9%; McNemar *p* = 0.11). Given a significant difference between age groups but no hemisphere difference, simple effect analyses examined group differences for each age group collapsing across hemisphere.

In younger subjects, collapsing across hemisphere, M100 responses were observed more often in TD (90%) than ASD + LI (66%; Fisher *p* = 0.04). No differences were observed between TD (90%) versus ASD − LI (76%; Fisher *p* = 0.14) or between ASD − LI (76%) and ASD + LI (66%; *p* = 0.53). In older subjects, collapsing across hemisphere, whereas no differences were observed between TD (91%) and ASD + LI (75%; Fisher *p* = 0.24), or between TD (91%) and ASD − LI (95%; Fisher *p* = 0.64), M100 responses were observed more often in ASD − LI (95%) than ASD + LI (75%; Fisher *p* = 0.03). Given similar differences in the presence/absence of M100 between TD versus ASD + LI and ASD − LI versus ASD + LI, the non-significant TD versus ASD + LI finding is likely due to a smaller *N* in the TD group. Splitting at the median head motion value, within each diagnostic age group, chi-square analyses showed that the presence or absence of M100 did not differ between individuals with more versus less head motion.

To assess if any of the missing M100 findings were specific to language impairment, the above analyses were re-run dividing the children with ASD into low and high PRI groups (median split). In younger subjects, collapsing across hemisphere, M100 responses were observed equally often in TD (88%) and PRI-high (78.2%; Fisher *p* = 0.29). Marginally significant differences were observed between TD (88%) versus PRI-low (70%; Fisher *p* = 0.07). No differences were observed between PRI-high (78.2%) and PRI-low (70%; *p* = 0.33). In older subjects, collapsing across hemisphere, no differences were observed between TD (91%) and PRI-high (91.3%; Fisher *p* = 1.0), between TD (91%) and PRI-low (86.4%; Fisher *p* = 0.70), or between PRI-high (91.3%) and PRI-low (86.4%; Fisher *p* = 0.69). Although not exactly the same, findings splitting children with ASD into low and high PRI groups were similar to findings splitting children with ASD into low and high CELF-4 groups. This is likely due to the fact that group membership remained largely unchanged. In particular, of the 25 ASD + LI subjects, 19 were in the lower half and 6 in the upper half of the PRI group, and of the 48 ASD − LI subjects, 9 were in the lower half and 39 in the upper half of PRI group.

Table 2 (middle column) shows the likelihood of observing a M100 for each group as a function of age (although when collapsing across group Fisher analyses showed a main effect of age, analyses show that at the group level a significant age differences was present only in ASD − LI). The Supplementary Material shows the likelihood of observing a M100 response in each group at each age.

### M200

Collapsing across group and hemisphere, M200 responses were observed equally often in older (94.7%) and younger subjects (98.3%; Fisher *p* = 0.13). Collapsing across group and age, the presence or absence of M200 did not differ between the right (96.4%) and left-hemisphere (97.8%; McNemar *p* = 0.69). Given no significant differences between age or hemisphere, simple effect analyses examined group differences for each group collapsing across age and hemisphere.

No differences were observed between TD (97.2%) and ASD + LI (94.7%; Fisher *p* = 0.68), TD (97.2%) and ASD − LI (98.4%; Fisher *p* = 0.62), or ASD − LI (98.4%) and ASD + LI (94.7%; Fisher *p* = 0.20).

Table 2 (right column) shows the likelihood of observing a M200 for each group as a function of age. The Supplementary Material shows the likelihood of observing a M200 response in each group at each age.

### M50 and M100 latency, hemisphere, and group

ANOVAs examined hemisphere and group latency differences in the subjects with a M50 and M100 response. For M50, simple effect analyses of a main effect of group, *F*(2, 107) = 4.59, *p* = 0.01, showed marginally earlier responses in TD (67 ms) versus ASD − LI (74 ms; *p* = 0.08) and significantly earlier responses in TD versus ASD + LI (77 ms; *p* = 0.01). The ASD groups did not differ (*p* = 0.74). The main effect of hemisphere, *F*(1, 107) = 0.11, *p* = 0.74, and the hemisphere by group interaction, *F*(2, 107) = 1.41, *p* = 0.25, were not significant. Findings were unchanged re-running analyses with age as a covariate. The pattern of findings was unchanged splitting the ASD group based on PRI, with the earliest M50 latencies in TD, second earliest in the PRI-high group, and the longest in the PRI-low group (PRI-low and PRI-high groups did not differ).

For M100, a main effect of hemisphere, *F*(1, 92) = 14.03, *p* < 0.001, showed earlier responses in the right (119 ms) than left (126 ms). There was no main effect of group, *F*(2, 92) = 1.42, *p* = 0.24. Although the interaction term was not significant, given the right-hemisphere TD versus ASD group latency findings in Roberts et al. (2010), *post hoc* analyses separately examined the left- and right-hemisphere. As expected, although group M100 latency differences were not observed in the left-hemisphere, *F*(1, 103) = 1.06, *p* = 0.31, a significant group M100 latency difference observed in the right-hemisphere, *F*(1, 92) = 4.87, *p* = 0.03, indicated earlier M100 latencies in TD (112 ms, SD 18.61) versus ASD (121 ms, SD 19.34). Findings were unchanged re-running analyses with age as a covariate. Analyses also showed M100 right-hemisphere latency differences between TD and the combined PRI group and no right-hemisphere M100 latency differences between ASD PRI-low and PRI-high.

Table 3 shows M50 and M100 latency mean and standard deviation values for each group and hemisphere.

**Table 3:** **Latency values in subjects with an observed M50 or M100**.

	*N* M50	M50 latency (ms)	*N* M100	M100 latency (ms)
		and SD		and SD
**CONTROLS**				
Left STG	33	67 (15)^a^	29	122 (27)
Right STG	32	66 (15)^a^	34	114 (22)^+^
**ASD − LI**				
Left STG	61	76 (18)	51	121 (27)
Right STG	54	72 (15)	53	121 (21)
**ASD + LI**				
Left STG	35	76 (17)	26	131 (25)
Right STG	31	79 (19)	29	121 (19)

*^a^ For M50, a main effect of group indicated earlier responses in TD versus ASD − LI and TD versus ASD + LI (*p* = 0.01). The ASD groups did not differ*.

*^+^Comparing TD to the combined ASD group, significant right-hemisphere M100 group differences were observed (*p* = 0.03)*.

### M50 and M100 latency and age

**Figure 1:**
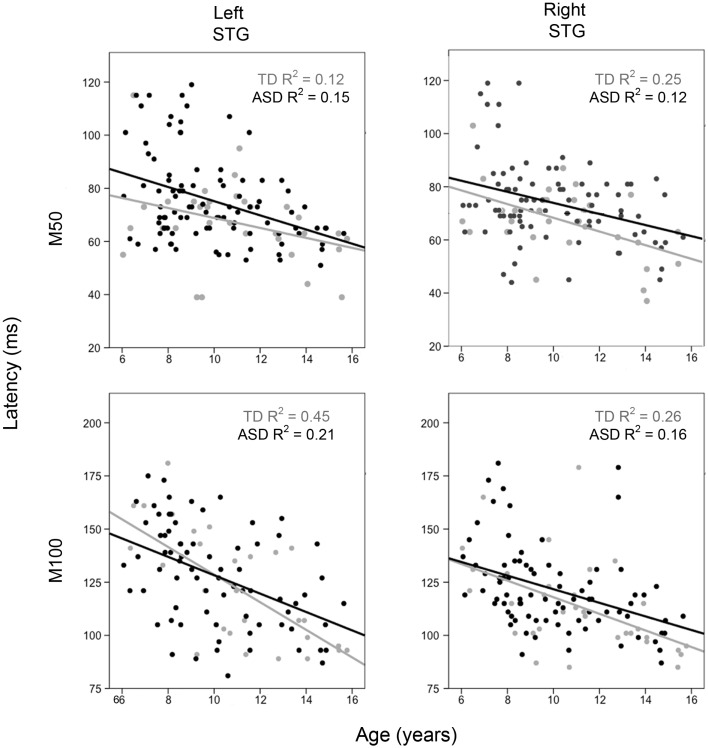
**Scatter plots showing associations between age and left and right M50 latency (upper row) and M100 latency (bottom row)**. Associations are shown for TD (light gray) and ASD (black). The *x* axis shows age and the *y* axis latency.

For each hemisphere, to examine how M50 and M100 latency differs as a function of group and age, hierarchical regressions were run in which age was entered first, diagnosis second, and their interaction last, with M50 or M100 latency as the dependent variable. For M50, in both hemispheres the full regression model (age, group, interaction) accounted for considerable variance in M50 latency (left = 18%; right = 18%, *p*’s < 0.001). Added first, age accounted for a significant 16% variance in left M50 latency (*p* < 0.001) and a significant 11% variance in right M50 latency (*p* < 0.001). Added second, group added a marginally significant 2% variance in left M50 latency (*p* = 0.06) and a significant 4% variance in right M50 latency (*p* < 0.05). The group × age interaction was not significant in either hemisphere (percent of variance <1%, ns). Figure 1 scatter plots show associations between age and left and right STG M50 latency (upper row).

For M100, in both hemispheres, the full regression model (age, group, interaction) accounted for considerable variance in M100 latency (left = 29%; right = 29%, *p*’s < 0.001). Added first, age accounted for a significant 28% variance in left M100 latency (*p* < 0.001) and a significant 25% variance in right M100 latency (*p* < 0.001). Added second, group added a non-significant 1% variance in left M100 latency (*p* > 0.05) and a marginally significant 3% variance in right M100 latency (*p* = 0.07). The group × age interaction was not significant in either hemisphere (percent of variance < 1%, ns). Figure 1 scatter plots show associations between age and left and right STG M100 latency (bottom row).

Given greater head motion in younger than older individuals, analyses were re-run using max head motion determined over the course of the scan as a covariate. Findings remained unchanged for all analyses.

### M50 and M100 latency and clinical measures

Regression analyses with PRI or CELF-4 scores entered first, diagnosis second, and their interaction last, with M50 or M100 latency as the dependent variable, showed no associations between the two clinical measures and M50 or M100 latency scores.

### Source time courses

**Figure 2:**
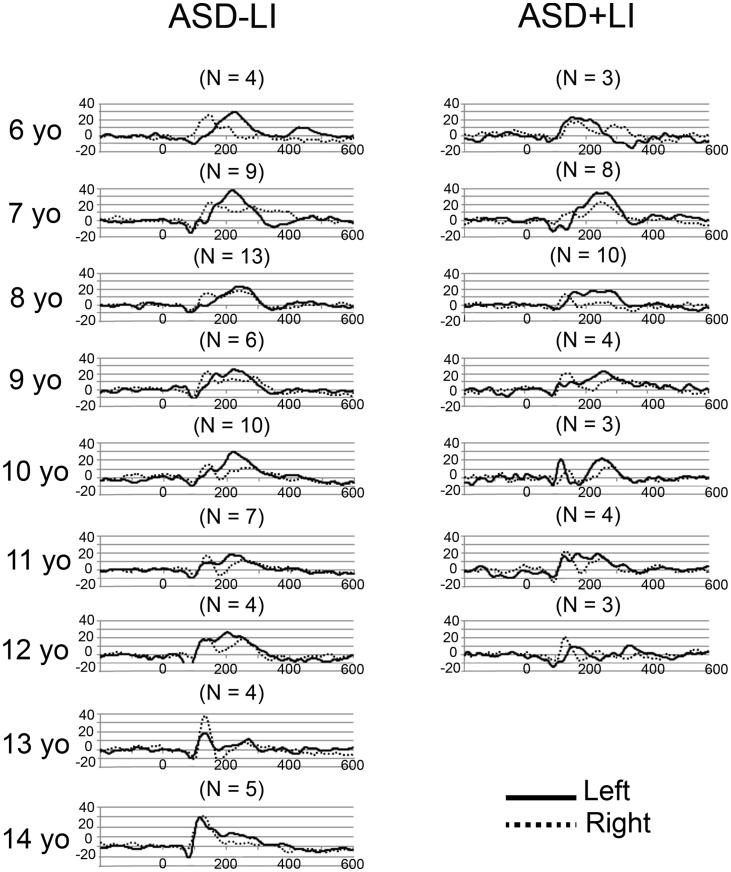
**Grand average left (solid) and right (dotted) STG source waveforms are shown for ASD as a function of age**. The *x* axis shows time and the *y* axis source strength. Given the small numbers in many of the age groups, grand average waveforms without standard errors are plotted to show general trends in each age group.

**Figure 3:**
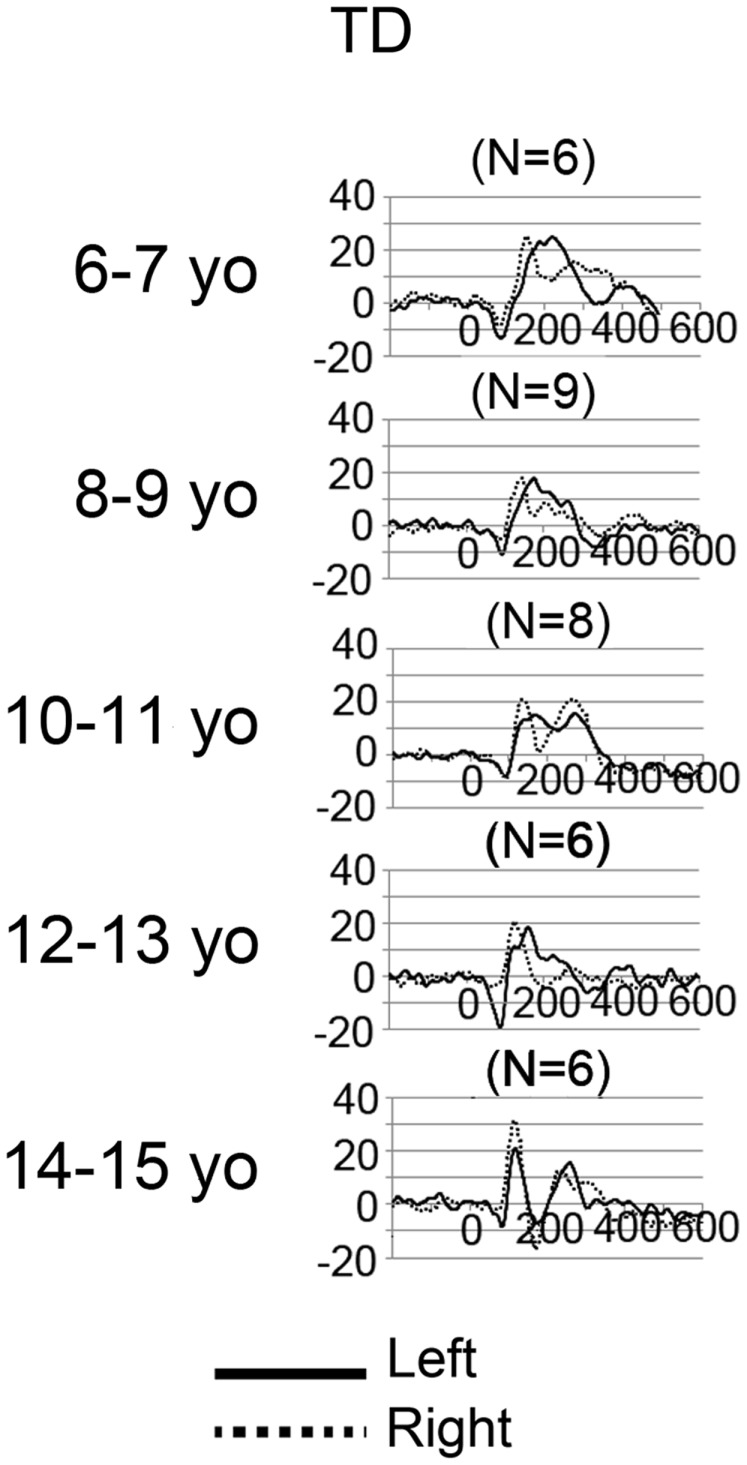
**Grand average left (solid) and right (dotted) STG source waveforms are shown for TD as a function of age**. The *x* axis shows time and the *y* axis source strength. Given the small numbers in many of the age groups, grand average waveforms without standard errors are plotted to show general trends in each age group.

Grand average left and right STG source waveforms are shown for ASD (Figure 2) and TD (Figure 3) as a function of age. Given a smaller *N* in the TD group (and thus fewer subjects at a specific age), whereas grand average waveforms are shown for each ASD by year age group, grand averages were computed in 2-year steps for TD.

Examination of the left (solid line) and right (dotted line) waveforms in the TD groups shows even in the youngest TD subjects a distinct left and right STG M100 (also indicated in the above chi-square and Fisher analyses). Examination of the ASD − LI and TD plots shows that the left STG M100 appears later in time (i.e., close to the M200 in younger subjects), and only develops into a clearly distinct component in older subjects. For example, only in the 14- to 15-year-old TD group is the left M100 peak clearly distinct from M200. This is in contrast to the right M100, where even in the 6- to 7-year-old TD group, M100 is distinct from M200. With regard to hemisphere differences, the source waveforms suggest that only in the oldest ASD − LI and TD subjects is there, on average, similarity in latencies between the two hemispheres.

## Discussion

### M50 and M100 abnormalities in ASD

Atypical auditory responses were observed in ASD. First, although STG M50 responses were observed in almost all subjects (replicating Ponton et al., 2002), M50 left and right responses were delayed by ~8 ms in ASD versus TD. Second, M100 responses were observed less often in ASD than TD; whereas a M100 response was observed in most younger and older TD controls, a M100 was missing in ~30% of the young and old ASD + LI subjects. Finally and perhaps unexpectedly, a significant age-dependent change in the presence of M100 was observed only in the ASD − LI subjects. As generally analogous findings were observed when dividing the ASD group by IQ, present findings cannot be uniquely interpreted as specific to language impairment and may instead be associated with general cognitive impairment.

In the present study, M50 responses were delayed bilaterally in children with ASD. This is in contrast to Roberts et al. (2010), where no M50 latency group differences were observed. Examination of Table 2 in Roberts et al. (2010), however, does show non-significantly later M50 latencies in the ASD than TD group for most frequencies. The difference between the previous and present findings is likely due to differences in the tasks (with a longer inter-trial interval in the present study) as well as greater power in the present study, with a twofold increase in TD subjects (17 versus 35) and a fourfold increase in ASD subjects (25 versus 101).

Other studies using similar paradigms (i.e., an auditory task with a long inter-trial interval), however, have reported different group latency findings. Using a paired-click paradigm and examining P50 responses at electrode Cz, Orekhova et al. (2008) observed a main effect (i.e., collapsing across the first and second click) of earlier P50 latencies in children with ASD versus TD (3- to 8-year-olds). Examination of Table 1 in Orekhova et al. indicates that the group P50 latency differences were most evident for the second click, with this perhaps accounting, in part, for study differences. Another difference is that whereas in the present study a high-pass filter of 1 Hz was applied, Orekhova et al. applied a 10 Hz high-pass filter. Kanno et al. (2000) and Yvert et al. (2001) have noted problems using a 10 Hz high-pass filter, showing in their studies that high-pass filters above 3 Hz produce artificial peaks in the P50 auditory response as well as distort the P50 electrical field topography. Finally, also using a paired-click task and examining P50 activity at electrode Cz, Oranje et al. (2013) observed non-significantly earlier P50 latencies in ASD versus TD (see Table 3). Given the above, additional studies are needed to replicate the present M50 group latency findings. In future studies, however, it is important to examine P50 activity in source rather than scalp space. As detailed in Edgar et al. (2003), given hemisphere differences in the strength, orientation, and latency of the left and right P50 auditory responses it is often not clear how to interpret findings associated with the multi-determined EEG Cz response (e.g., a response reflecting activity from, at least, left and right STG).

Whereas a delayed M50 could be considered a risk factor for ASD, present findings suggest that a missing M100 in younger children with ASD is a risk factor for language impairment, and in older ASD children a missing M100 is a marker for ASD with concomitant language impairment. It is important to note, however, that in addition to lower CELF-4 scores, the ASD + LI group also had lower PRI scores (a non-verbal IQ measure that minimizes emphasis on verbal skills) than the TD and ASD − LI groups. Thus, lower cognitive ability rather than language impairment likely account for the present findings and thus the hypothesis that language impairment would best predict missing M100 responses was not supported. Finally, *post hoc* analyses examining only the right-hemisphere showed significant right-hemisphere M100 latency delays in ASD + LI and ASD − LI versus TD (see Table 3). Thus, as in Roberts et al. (2010), present findings suggest that a delayed right-hemisphere M100 is common to ASD with and without language impairment.

The above M50 and M100 abnormalities in ASD may reflect distinct auditory cortex abnormalities. Development of deep layers (lower layer III to layer VI) in auditory cortex occurs between 6 months and 5 years of age (e.g., Ponton et al., 1999). In contrast, the superficial layers (upper layer III and layer II) continue to mature until about age 12 (Moore and Guan, 2001; Moore and Linthicum, 2007). Based on this, these researchers have hypothesized that the 50 ms auditory response reflects recurrent activation in layers III and IV, the termination zone of thalamo-cortical pathways that are almost fully developed in by age 6. M100, however, is observed less frequently in young children as generation of M100 likely reflects activation of cortical layers upper III and II, areas not fully developed until age 12 (e.g., Ponton et al., 1999, 2002; Ponton and Eggermont, 2001).

In the present study, observation of a M50 response in all groups likely reflects development of cortical layers III and IV, with the delayed M50 latency in both ASD groups perhaps indicating slower maturation of these layers, perhaps due to delayed myelination of thalamo-cortical pathways in ASD (Roberts et al., 2009, 2013). Lack of a M100 in the young ASD − LI and ASD + LI groups may indicate delayed maturation of layer II and upper layer III in ASD with abnormally decreased synchronization of afferent activity arriving at the synapses in layer II and upper layer III resulting in a greatly desynchronized M100 (Ponton and Eggermont, 2001). Whereas the finding of a missing M100 in younger but not older ASD − LI suggests delayed but continued maturation of layer II and upper layer III in ASD − LI, the lack of M100 in some older ASD + LI subjects indicates a more profound disruption of secondary auditory cortex areas in these subjects, perhaps never developing a fully functional set of superficial layer axons.

It may be, however, that M50 latency delays in ASD solely reflect M100 abnormalities. As reviewed in the Section “Introduction,” Ponton et al. (2002) noted that during development, the magnitude of the earlier maturing tangential “50 ms” auditory response decreases as the magnitude of the later maturing tangential “100 ms” auditory response increases. Given this pattern, in the present study, whereas in the TD group a normally developing M100 responses could result in an earlier M50 response via “cancelation” of M50 activity via a increasingly dominant M100 response, in ASD a missing or abnormally developing M100 response could result in less “cancelation” of the M50 response and thus longer M50 latencies (even in the absence of abnormalities in layers III and IV). Longitudinal rather than cross-sectional studies are needed to understand the development of P50/M50 and N100/M100 auditory responses in order to better understand the present findings.

In any case, as described in Moore and Guan (2001), disruption in the development of STG auditory areas between the ages of 5 and 12 would lead to a failure to develop a meshwork of vertical and horizontal axons in the superficial layers, with axons in the superficial layer representing primarily (though not exclusively) corticocortical projections. Such disruptions could account for temporal lobe resting state abnormalities (Cornew et al., 2012) as well as the STG abnormalities observed in the present study. Given the possibility of a cascading effect, with early abnormalities increasingly distorting (or not allowing) normal development of upper cortical layers in some individuals with ASD, similar to the recommendations suggested for children with hearing impairments (e.g., Kral and Eggermont, 2007; Moore and Linthicum, 2007), present findings indicate the need for early treatment in ASD to increase the chance of normal development of auditory areas throughout childhood and adolescence.

### M50 and M100 latency associations with age

Replicating previous studies (see Introduction), M50 and M100 latency decreased as a function of age. Present findings are also consistent with previous studies showing that the M100 develops “out of” the M200 response (Ponton et al., 1999, 2002), with present findings also indicating that the development of M100 as a component distinct from M200 occurs more slowly in the left-hemisphere. Studies examining hemisphere differences in even younger subjects are of interest to more fully understand the development of M100.

Although as noted in the Section “Introduction,” there is strong evidence for a decrease in P50 latency as a function of age, using a paired-click paradigm similar to the paradigm used in the present study, age-related P50 latency findings using this paradigm have been mixed. Using the standard auditory paired-click paradigm (i.e., 500 ms ISI) and examining individuals from 1 to 65 years, Freedman et al. (1987) observed a negative correlation between Cz P50 latency and age for the entire population, with a rapid change in latency observed in children aged 1–8 years. Myles-Worsley et al. (1996) also observed earlier P50 latencies in a younger (10–14 years) versus older groups (15–19, 20–29, and 30–39 years). However, also using the standard paired-click paradigm and examining children, Rasco et al. (2000), Marshall et al. (2004), and Brinkman and Stauder (2007) did not observe associations between P50 latency and age. Similar to the previously noted ASD P50 Cz studies, a lack of an age-related P50 latency findings in Cz paired-click studies may be due to the examination of the multi-determined scalp P50 response (left and right STG activity) as well as in some of these studies the potentially problematic use of a 10 Hz high-pass filter to examine P50.

### Limitations and future directions

A potential limitation of the present study is that although the orientation of the STG dipoles was optimized for each subject, the STG dipoles were placed at standard locations rather than localized for each subject. This was necessary in the present study as M50 and/or M100 responses were not observed in some subjects; thus use of a standard source model allowed assessment of the primary question in the present study – determining the presence or absence of a left or right M50 and M100 STG response in children and adolescents with ASD.

As shown via examples in the Supplementary Material, although the estimated strength of the M50 and M100 response in some subjects could be inaccurate when using a standard source model, it is not likely that the latency estimate is inaccurate. In addition, present findings also show that in younger subjects M100 overlaps with M200. As such, whereas in young subjects localization of M100 would primarily reflect M200 generators, in older subjects M100 would reflect only M100 generators. Thus, in the present study, use of a standard model was not only necessary but also sufficient to examine the study questions.

Further considering the use of a standard model, present findings demonstrate a dilemma in this area of research: when examining M100 activity in individuals younger than ~13 years old, it is probably not possible to empirically determine whether localization of M100 in each subject provides more accurate information than the information provided by a standard source model (given the overlap between M100 and M200). Present findings also clearly indicate that developmental studies examining N100 activity at a single midline scalp site (e.g., Cz or Fz) are problematic as latency and amplitude measures at a single site in any individual could reflect activity from only a single hemisphere or, more likely, from some *a priori* unknown combination of M100 and M200 activity from each hemisphere. Indeed, Ponton et al. (2002) and Sussman et al. (2008) note that examining sensor data is problematic as the activity at any given sensor location reflects the weighted contribution of activity from different sources, each with potentially different maturation rates. Present findings suggest, however, that in older ASD − LI and TD subjects a single electrode EEG measure could be sufficient, as Figures 2 and 3 suggest greater similarity in the left and right waveforms in these older subjects [although see Edgar et al. (2003) for a more detailed discussion of these issues].

It is possible that present findings could be improved via co-registering each subject to their own MRI and then using anatomical constraints (e.g., identifying each individuals left and right Heschl’s gyrus) to place dipoles, a strategy that will be examined in future studies. Although possible in older children, as it is often not possible to obtain structural MR data in infants and young children, for some studies it will still be necessary to apply techniques to align MEG data to template MRIs. In future studies examining younger children, the use of whole-brain infant and young child MEG systems will be preferred, with a smaller helmet size providing optimal signal-to-noise in younger children (Roberts et al., 2014).

Other limitations are of note. First, although MEG provides excellent assessment of auditory activity on the surface of STG (i.e., tangential auditory activity), MEG does not easily detect radial sources. As such, studies using EEG (or simultaneous EEG + MEG) are needed to examine radially oriented STG auditory sources (e.g., see Ponton et al., 2002; Stroganova et al., 2013). Second, studies examining auditory responses in ASD using non-passive tasks are needed to assess the generalizability of the present findings. Third, in the present study, in some subjects, it was difficult to identify the M100 response with 100% certainty, especially in younger subjects where M100 just emerges from M200. As an example, as shown in Figure 3 (TD subjects), the right STG grand average waveforms (solid black) for the 6- and 7-year-olds show what could be a single M200 response, or a M200 preceded by a M100. In contrast, in Figure 3, the right STG grand average waveforms for the 10- and 11-year-olds show what is very clearly identified as a M100 followed by a M200. The source waveforms show that in young subjects it is intrinsically difficult to determine whether M100 is truly distinct from M200. Thus, in young ASD subjects, “follow-up” exams may be needed to monitor the development (or lack of development) of an M100 response. In the present study, M100 was scored as present if there was a peak with a rising and falling slope distinct from the M200, with a M100 magnetic-field topography, and with a latency between 80 and 185 ms. In the present study, in the few cases of ambiguous M100 determination, the final dichotomous assignment was determined by consensus review.

Finally, present findings as well as other studies indicate the need for longitudinal studies to more fully understand the development of auditory responses. For example, it has been reported that in some subjects, two M50 responses are observed. For example, using source modeling to examine left and right STG activity in children aged 7- to 16-years-old, Orekhova et al. (2013) observed in many subjects two components preceding the M100 response; a relatively low-amplitude response at ~65 ms (observed in ~50% of the subjects in the left-hemisphere and 75% of the subjects in the right-hemisphere) and a much more prominent later response with a M50 topography at ~100 ms. In the present study, M50 was defined as the first field reversal preceding M100 (or M200 if M100 not present). Although in the present study examination of the M50 latencies did not reveal a bi-modal distribution, additional longitudinal studies are needed to more fully examine the development of P50/M50 response(s).

## Conclusion

Although almost all TD and ASD subjects had a M50 response, M50 responses were delayed in ASD than TD bilaterally. Although M100 latencies were longer in the left- than right-hemisphere in TD, this delay was not so pronounced such that even young TD subjects had an identifiable left and right M100 by 6 years of age. Whereas there was a significant increase in the presence of M100 responses in the older than younger ASD − LI group, many individuals in the older ASD + LI group had a missing M100. Examining the TD data, present findings indicate that by 11 years, a right M100 should be observed in 100% of subjects and a left M100 in 80% of subjects. Thus, by 11 years old, if a long inter-trial interval is used, lack of a left and especially right M100 offers neurobiological insight into abnormal sensory processing that may underlie language or cognitive impairment in ASD.

## Conflict of Interest Statement

The Reviewer Elysa Jill Marco declares that despite having collaborated with the author Timothy P. L. Roberts on another project, the review process was handled objectively and no conflict of interest exists. The authors declare that the research was conducted in the absence of any commercial or financial relationships that could be construed as a potential conflict of interest.

## Supplementary Material

The Supplementary Material for this article can be found online at http://www.frontiersin.org/Journal/10.3389/fnhum.2014.00417/abstract

## Abbreviations

ASD − LI, autism spectrum disorders without language impairment; ASD + LI, autism spectrum disorders with language impairment; CELF-4, clinical evaluation of language fundamentals – fourth edition; CHOP, Children’s Hospital of Philadelphia; EEG, electroencephalography; MEG, magnetoencephalography; PRI, perceptual reasoning index; STG, superior temporal gyrus; TD, typically developing; VCI, verbal comprehension index.
