# Supplementary material for: Missing and Delayed Auditory Responses in Young and Older Children with Autism Spectrum Disorders
Source: Front Hum Neurosci. 2014 Jun 6;8:417. doi: 10.3389/fnhum.2014.00417 (PMC4047517; doi:10.3389/fnhum.2014.00417)
Supplement: Supplementary file 1 [file Presentation1.PDF]

ONLINE SUPPLEMENT

Supplement Table 1: Likelihood of observing a M50, M100, and M200 for each group by age (two-year intervals) for the left hemisphere (left), right hemisphere (right), and the average of the left and right hemisphere.

M50

**TD**	N	Left	Right	Average
6-7yrs	6	100.0%	100.0%	100.0%
8-9yrs	9	90.0%	90.0%	90.0%
10-11yrs	8	87.5%	100.0%	93.8%
12-13yrs	6	100.0%	66.7%	83.3%
14-15yrs	6	83.3%	83.3%	83.3%
**ASD-LI**				
6-7yrs	13	92.3%	84.6%	88.5%
8-9yrs	19	94.7%	84.2%	89.5%
10-11yrs	17	100.0%	76.5%	88.3%
12-13yrs	8	100.0%	100.0%	100.0%
14-15yrs	6	100.0%	100.0%	100.0%
**ASD+LI**				
6-7yrs	11	100.0%	90.9%	95.5%
8-9yrs	14	92.9%	71.4%	82.2%
10-11yrs	7	85.7%	71.4%	78.6%
12-13yrs	4	100.0%	100.0%	100.0%
14-15yrs	2	50.0%	100.0%	75.0%

M100

**TD**	N	Left	Right	Average
6-7yrs	6	83.3%	100.0%	91.7%
8-9yrs	9	77.8%	88.9%	83.3%

10-11yrs	8	87.5%	100.0%	93.8%
12-13yrs	6	83.3%	100.0%	91.7%
14-15yrs	6	83.3%	100.0%	91.7%
**ASD-LI**				
6-7yrs	13	69.2%	84.6%	76.9%
8-9yrs	19	78.9%	78.9%	78.9%
10-11yrs	17	76.5%	76.5%	76.5%
12-13yrs	8	100.0%	100.0%	100.0%
14-15yrs	6	100.0%	100.0%	100.0%
**ASD+LI**				
6-7yrs	11	63.6%	72.7%	68.2%
8-9yrs	14	71.4%	78.6%	75.0%
10-11yrs	7	85.7%	71.4%	78.6%
12-13yrs	4	50.0%	75.0%	62.5%
14-15yrs	2	50.0%	100.0%	75.0%

M200

**TD**	N	Left	Right	Average
6-7yrs	6	100.0%	100.0%	100.0%
8-9yrs	9	100.0%	100.0%	100.0%
10-11yrs	8	100.0%	100.0%	100.0%
12-13yrs	6	83.3%	83.3%	83.3%
14-15yrs	6	100.0%	100.0%	100.0%
**ASD-LI**				
6-7yrs	13	100.0%	100.0%	100.0%
8-9yrs	19	100.0%	94.7%	97.4%
10-11yrs	17	100.0%	94.1%	97.1%
12-13yrs	8	100.0%	100.0%	100.0%
14-15yrs	6	100.0%	100.0%	100.0%
**ASD+LI**				
6-7yrs	11	100.0%	100.0%	100.0%
8-9yrs	14	100.0%	92.9%	96.5%
10-11yrs	7	100.0%	100.0%	100.0%
12-13yrs	4	75.0%	75.0%	75.0%
14-15yrs	2	50.0%	100.0%	75.0%

Accuracy of M100 Latency Measures

Analyses examined (1) how localization errors introduced by use of a standard model affect M100 latency measures, (2) how M100 latency measures change as a function of the location of the standard STG M100 source, and (3) if there was evidence determining the presence/absence of a missing M100 via examination of the superior temporal gyrus (STG) source waveforms missed M100 responses by comparing left and right STG waveforms and responses in the left and right MEG sensors.

<u>Comparing measures obtained via standard source model and via individual source localization</u>

1. In eleven healthy adults, left and right superior temporal gyrus M100 latencies were obtained using a standard source model (i.e., as in the paper) and using individual source

localization. Using these two methods, left and right STG M100 latency measures in response to tones of 200, 300, 500, and 1000 Hz sine waves were obtained and compared.

Left and right STG M100 latencies using a standard source model were determined using the procedures outlined in the paper. For individual source localization, the data were filtered (trial averages) using two fourth order zero-phase elliptical filters (0.5dB peak-to-peak ripple, 60dB stopband attenuation) with (i) a high-pass filter with a cutoff of 1 Hz and (ii) a low-pass filter with a cutoff of 40 Hz. After filtering and baseline correction (over the entire 0.5 sec pre- and post-trigger epoch), the root-mean-square (RMS) per hemisphere was calculated (using all available channels), and the M100 latency measure obtained; this analysis utilized MATLAB R2009a(The Mathworks 2009) and FieldTrip (Oostenveld et al. 2011) to read in and obtain the M100 sensor-level measurements. Verification of the M100 RMS latency measure was performed using (i) dipole fit estimates using VSM MedTech proprietary software and (ii) examination of the sink-source distribution of the left and right M100 magnetic fields. Any responses that did not localize to a reasonable location (e.g., close to the temporal lobe) or did not match the canonical M100 source-sink distribution were removed; a total of sixteen such deletions were made.

To assess associations between the standard and individual M100 latency measures, three statistical tests were performed using R 2.14.1(R Development Core Team 2011): (i) Spearman's rank correlation; (ii) Kendall's rank correlation and (iii) a Kolmogorov-Smirnov test. As shown in Online Supplement Figure 1, across hemisphere and frequency, the standard and individual latency measurements were highly correlated (Spearman: $\rho = 0.94$, $S = 855.79$, $p < 0.001$; Kendall: $\tau = 0.82$, $z = 7.78$, $p < 0.001$). The Kolmogorov-Smirnov test indicated that the latency distributions were not significantly different from one another ($D = 0.20$, $p = 0.17$).

Online Supplement Figure 1

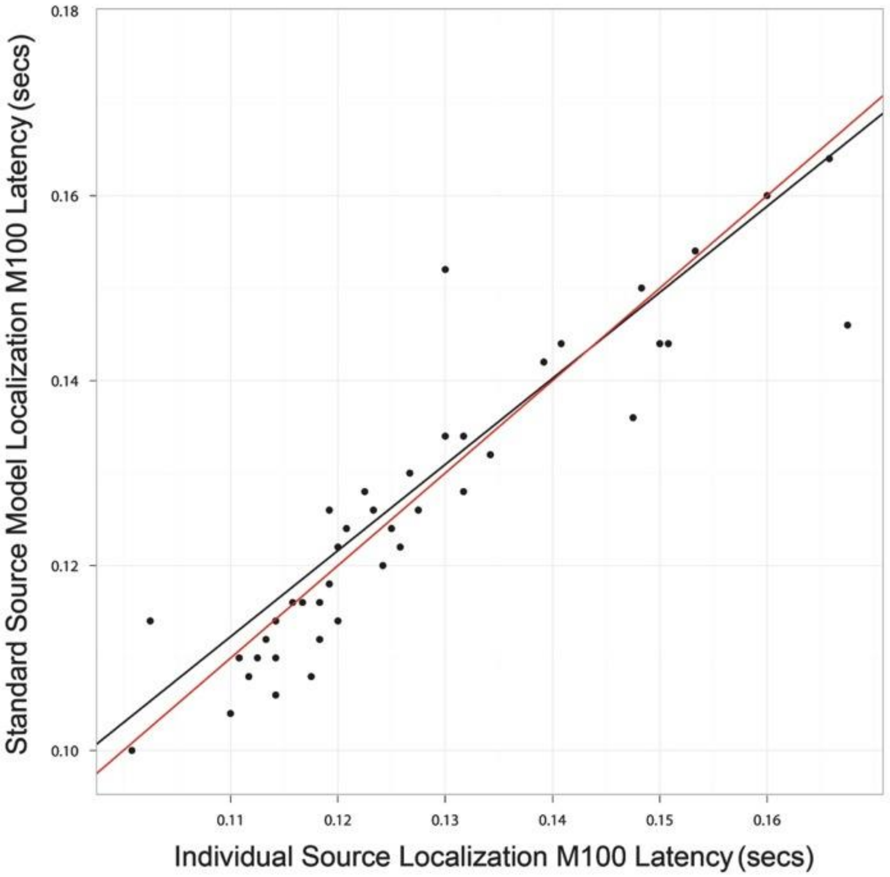

Figure caption: Online Supplement Figure 1 illustrates the relationship between M100 latency measurements obtained using both evaluation methods. Latency measurements based on individual source models are on the x axis; measurements based on a standard source model are on the y axis. A line of best fit for the data (intercept = 0.009, slope = 0.93) is illustrated by the sold black line; for comparison, a line with unity slope and an intercept of zero is superimposed in red.

<u>Examining error: Standard source model</u>

In 2 subjects, the STG source waveform (3000 ms ISI) obtained using the standard source model (i.e., with the dipole oriented at M100) was compared to source waveforms where the dipole was moved with respect to the standard location (1) 1 cm anterior, 1 cm lateral, and 1 cm superior,

(2) 1 cm posterior, 1 cm media, and 1 cm inferior, (3) 1 cm anterior, 1 cm medial, and 1 cm superior, and (4) 1 cm posterior, 1 cm lateral, and 1 cm inferior.

As shown in Online Supplement Figure 2, although M100 source strength changes as a function of depth (more medial sources stronger, more lateral sources weaker), moderately large errors in source localization did not affect M100 latency measure. These data thus suggest that any errors in M100 localization are unlikely to account for the study findings.

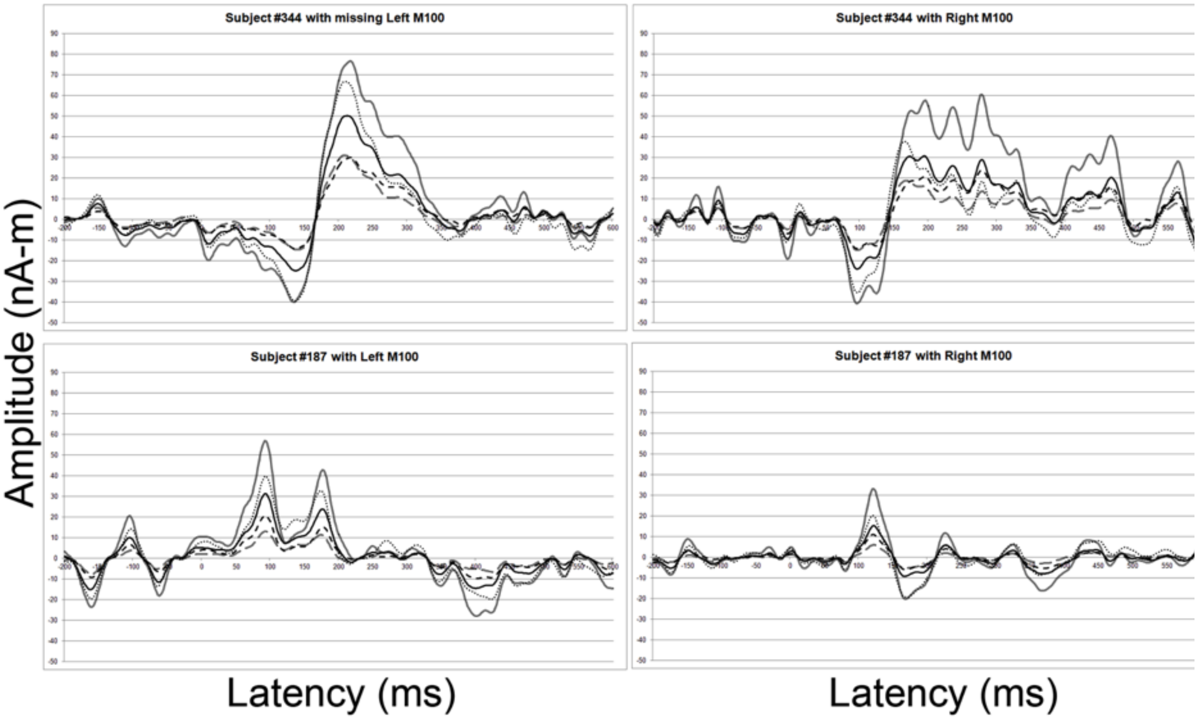

Figure 2: Shows changes in source waveforms as a function of deviation from standard location (show in solid black). Subject #344 (top row) is a 7-year-old ASD-LI subject with a missing left M100 and a present right M100. Note that in the left hemisphere there is no indication that a recognizable M100 appears at any of the ‘deviant’ locations. Subject #187 (bottom row) is a 13-year-old TD subject with M100 present in both hemispheres. Note that although the strength of the M100 changes as a function of location, in no case does the latency change.

<u>Examining MEG sensors in individuals with missing M100 responses</u>

STG source waveform (3000 ms ISI) and MEG sensor data is shown for 4 subjects. Examination of the sensor waveforms in individuals with a missing M100 does not indicate a M100 present in the MEG sensors but missing in the STG source waveforms.

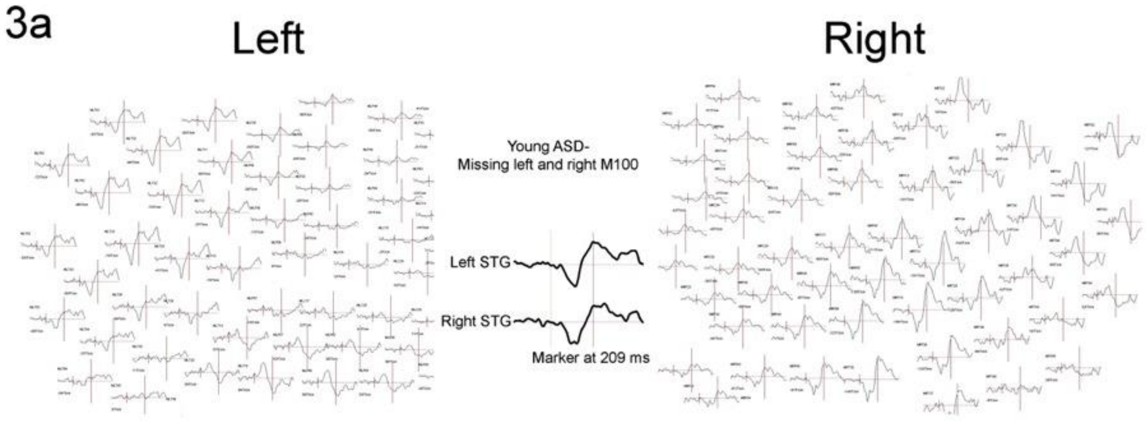

Figure 3a shows data from a young ASD- subject with missing left and right M100 responses. Although a M50 and M200 are clearly observed in both the left and right source and sensor waveforms, a M100 is not observed.

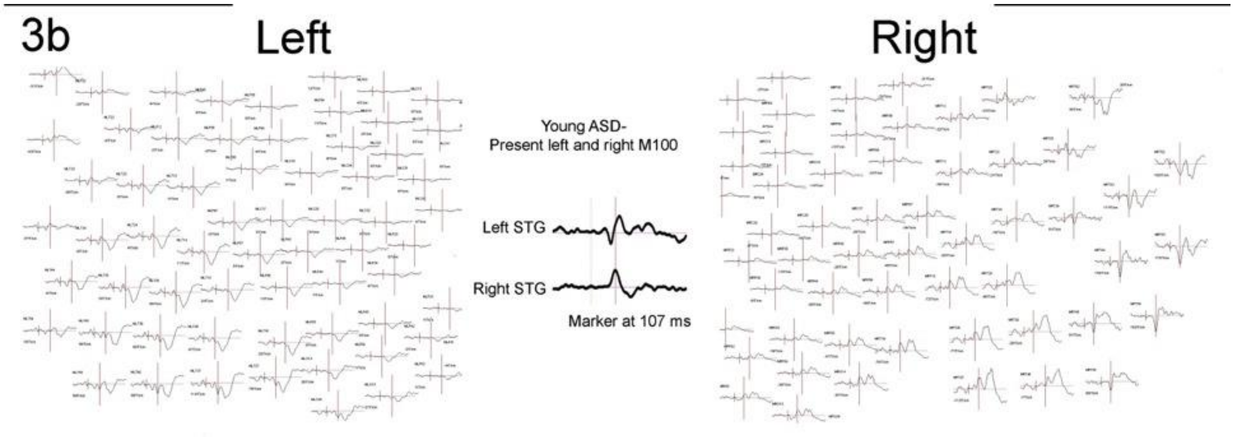

Figure 3b shows data from a young ASD- subject with M100 responses in the left and right hemisphere. M100 responses are seen in the source waveform and sensor data. This subject shows a typical latency pattern, with an earlier M100 response in the right than left.

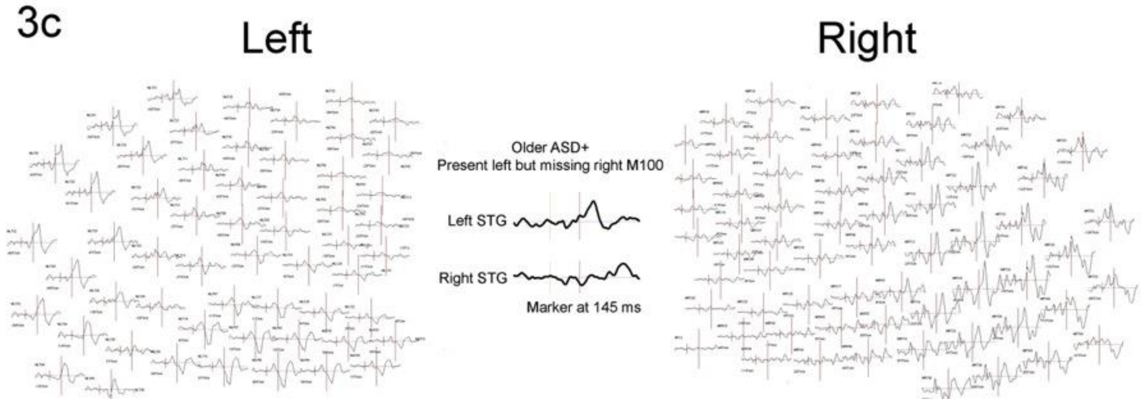

Figure 3c shows data from an older ASD+ subject with a present left M100 but a missing right M100. Notice that the right M100 at 145 ms is seen to develop ‘out’ of the later M200.

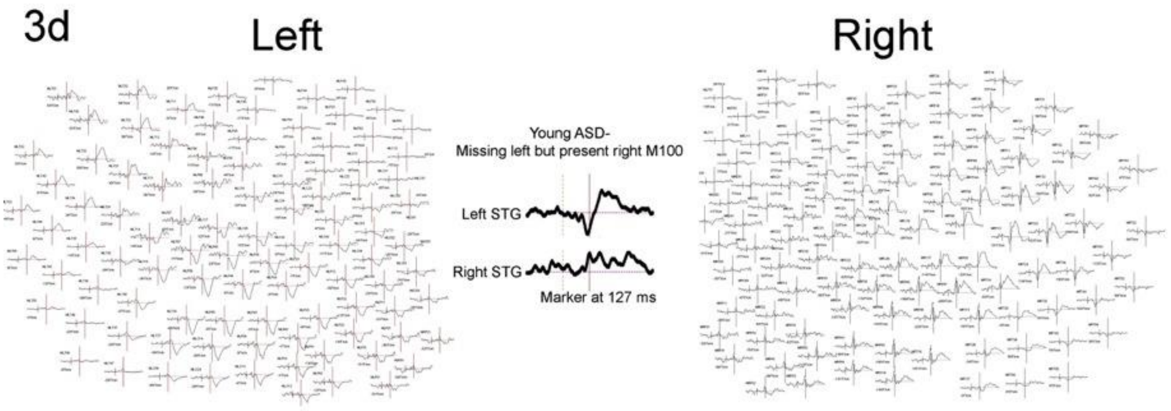

Figure 3d shows data from a young ASD- with a missing left M100 but a present right M100. Although a M50 and M200 are clearly observed in both the left source and sensor waveforms, a M100 is not observed.

Online Supplement References

The Mathworks . MATLAB Version R2009a. Natick, MA: The Mathworks, 2009.

Oostenveld R, Fries P, Maris E, and Schoffelen JM. FieldTrip: Open source software for advanced analysis of MEG, EEG, and invasive electrophysiological data. *Comput Intell Neurosci* 2011: 156869, 2011.

R Development Core Team. R: A language and environment for statistical computing. R 2.14.1. Vienna, Austria: R Foundation for Statistical Computing, 2011.
